# A comparative analysis of metabolic and urinary bacterial culture profiles between patients with staghorn and non-staghorn infectious renal stones

**DOI:** 10.1186/s12882-026-05204-5

**Published:** 2026-07-17

**Authors:** Endi Zhang, Zhichao Chi, Xuming Zhang, Jianxing Li, Chaoyue Ji, Weiguo Hu

**Affiliations:** Department of Urology, Beijing Tsinghua Changgung Hospital, School of Clinical Medicine, Tsinghua Medicine, Tsinghua University, Beijing, 102218 China

**Keywords:** Infectious stones, Staghorn calculi, Serum calcium, Metabolic analysis, 24-hour urine, Urine bacterial culture

## Abstract

**Objective:**

To investigate the risk factors of staghorn calculus and explore the differences in the distribution of culturable urine bacteria among patients with infectious renal stones.

**Methods:**

A retrospective analysis was conducted on the clinical data of 201 patients hospitalized in our center from January 2020 to December 2024. Patients who underwent upper urinary tract stone surgery and whose postoperative stone composition analysis indicated that the main component was infectious stones were included. The patients were divided into two groups based on the presence or absence of staghorn calculi. General data, serum biochemical parameters, 24-hour urine metabolic parameters, and urine culture results were collected and compared between the two groups. Univariate and multivariate logistic regression analyses were conducted to identify independent risk factors for staghorn calculus.

**Results:**

The two groups revealed no significant differences in terms of gender, age, or comorbidities. Serum calcium levels (*P* < 0.05), estimated glomerular filtration rate (eGFR) (*P* < 0.05), and 24-hour urinary calcium levels (*P* = 0.003) were significantly higher in the non-staghorn stone group than in the staghorn stone group. Multivariate logistic regression analysis indicated that lower serum calcium levels were independently associated with staghorn calculus formation (*P* = 0.025, OR 0.032, 95% CI [0.002, 0.649]). Urine culture analysis revealed that the detection rate of *Proteus mirabilis* was significantly higher in the staghorn stone group than in the non-staghorn stone group (31.5% vs. 2.4%, *P* = 0.001).

**Conclusion:**

This study suggests that lower serum calcium levels and *Proteus mirabilis* infection are associated with staghorn calculi in patients with infectious stones. Clinically, monitoring serum calcium levels and managing urinary tract infections may help reduce the risk of staghorn calculus progression and preserve renal function. However, these findings require further validation through prospective studies.

## Introduction

Kidney stones represent a common urological disorder, whose incidence has been steadily increasing over the past three decades and currently affects approximately 10% of the global population [1, 2]. Characterized by high incidence, high recurrence rates, and high hospitalization rates, kidney stones impose a substantial burden on global healthcare systems [3]. Healthcare expenditure on urolithiasis in the United States alone reached $5.3 billion as early as 2014. Given the ongoing trends in the prevalence of urolithiasis, this will undoubtedly continue to strain local healthcare systems [4].

Infectious stones are a specific type of urinary calculus, accounting for nearly 15% of all cases. These stones are primarily composed of struvite (magnesium ammonium phosphate hexahydrate), carbonate apatite, and ammonium acid urate [5, 6]. The formation of infectious stones is closely associated with urinary tract infections caused by urease-producing bacteria [7, 8]. Under the action of the enzyme urease, urea is hydrolyzed into ammonia and carbon dioxide. This leads to the formation of ammonium ions and increases urine pH, establishing alkaline conditions. These two conditions promote the crystallization of struvite and carbonate apatite [5, 9]. A notable characteristic of infectious stones is their rapid growth, which readily leads to the formation of staghorn calculi [10]. Depending on the extent of involvement of the renal calyces, staghorn calculi can be classified as complete or partial.

Staghorn calculi are typically associated with infections and progressive renal impairment [11], and often necessitate prolonged surgical procedures with a high risk of multiple postoperative complications, making their management challenging [11]. Although numerous studies have focused on staghorn calculi, a few have compared the metabolic profiles and distributions of culturable urine bacteria between patients with infectious staghorn stones and those with non-staghorn infectious stones. Furthermore, although infectious stones are prone to progress to staghorn calculi, not all patients with infectious stones exhibit this characteristic morphology. This suggests potential differences in the underlying mechanisms and metabolic factors, which may finally affect clinical treatment and prevention strategies. Therefore, this study retrospectively analyzed data from patients with infectious stones who were hospitalized in our center between January 2020 and December 2024. General demographic characteristics, serum biochemical parameters, and 24-hour urine metabolic indicators were compared between the staghorn and non-staghorn stone groups to identify the factors affecting staghorn stone formation in patients with infectious stones. This study aimed to provide a theoretical basis for preventing staghorn calculus formation in this population.

## Materials and methods

### Study patients

A retrospective analysis was conducted on the clinical data of patients who were diagnosed with infectious stones and underwent upper urinary tract stone surgery at our center between January 2020 and December 2024. The composition of all stones was determined using Fourier transform infrared spectroscopy. Patients whose infectious stone components (magnesium ammonium phosphate hexahydrate, carbonate apatite) accounted for ≥ 50% of the total stone composition were included in this study. The exclusion criteria were as follows: (1) concurrent anatomical abnormalities of the urinary tract(e.g., horseshoe kidney, ectopic kidney, transplanted kidney) or urinary tract dysfunction (e.g., neurogenic bladder); (2) patients on dialysis; (3) history of antibiotic use within 7 days prior to admission; and (4) patients receiving oral calcium supplementation in the long term. This study was approved by the Ethics Committee of Beijing Tsinghua Changgung Hospital, Tsinghua University.

### Research method

Baseline demographic data (gender and age), blood pressure, body mass index (BMI), comorbidities (e.g., hypertension and diabetes), preoperative laboratory data (serum biochemistry, 24-hour urine, and urine culture), and related imaging data were collected and organized. Hypertension was diagnosed following the 2020 International Society of Hypertension Guidelines for the Practice of Hypertension Management [12], and diabetes was diagnosed following the 2021 American Diabetes Association Classification and Diagnosis Criteria for Diabetes [13]. Based on the results of computed tomography (CT), plain radiography of the kidney, ureter, and bladder (KUB), and renal ultrasound, patients were divided into the staghorn calculi group and the non-staghorn calculi group. If patients had multiple medical records, only data from their first visit were used for statistical analysis.

### Midstream urine specimen collection

All enrolled patients were hospitalized prior to surgery. After admission and before antibiotic therapy and any intervention, 10 mL of random midstream urine was collected using a sterile collection tube and immediately sent for microbiological culture. If immediate testing was not possible, the specimen was kept at 4 °C and sent for analysis within 8 h. This ensured that the laboratory test results reflected the baseline clinical status and that the urine culture results were not affected by antibiotic use.

### Blood biochemical analysis and 24-hour urine collection

To ensure measurement accuracy, patients fasted before morning venous blood collection. Routine biochemical tests were conducted to measure blood electrolytes, urea nitrogen, creatinine, uric acid, parathyroid hormone (PTH), and estimated glomerular filtration rate (eGFR). 24-hour urine collection was conducted from 8:00 AM on the second day of hospitalization until 8:00 AM the following day. Hydrochloric acid was added as a preservative to stabilize urine components during sample collection. Urine analysis was conducted using certified automated analyzers following standardized methods. Urine pH, total urine volume, calcium, phosphorus, sodium, potassium, chloride, and uric acid excretion rate were measured during urine analysis. Throughout the collection period, patients are instructed to avoid strenuous physical activity and maintain detailed dietary records.

### Statistical analysis

Statistical analysis was conducted using SPSS software version 27.0. Categorical data are expressed as the number of cases (N) and the corresponding percentage (%). Normally distributed continuous data are presented as mean ± standard deviation (SD), and between-group comparisons were conducted using the Chi-square test, t-test, and Fisher’s exact test. Non-normally distributed continuous data are expressed as median (Q1, Q3), and the Mann-Whitney U test was applied for intergroup comparisons. Multivariate logistic regression analysis was employed to identify independent risk factors for staghorn calculus formation in patients with infectious stones. Variables with *P* < 0.10 in univariate analysis, together with clinically relevant variables, were considered candidates for inclusion in the multivariable model. Prior to model construction, multicollinearity was assessed using variance inflation factors (VIFs) and tolerance values. Variables with VIF > 5 or tolerance < 0.2 were considered to indicate potentially problematic collinearity. Continuous variables were entered into the regression model using their original measurement units and were not standardized. Odds ratios (ORs) and corresponding 95% confidence intervals (CIs) were calculated. A two-sided P value < 0.05 was considered statistically significant. A P-value < 0.05 was deemed statistically significant.

## Results

### Demographic and baseline characteristics of patients

In total, 201 patients with infectious stones were included in this study (Table 1). The staghorn calculi group comprised 130 cases (64.7%), including 41 males (20.4%) and 89 females (44.3%), with a mean age of 52.1 ± 11.4 years. The non-staghorn calculi group consisted of 71 cases (35.3%), including 22 males (10.9%) and 49 females (24.4%), with a mean age of 49.1 ± 13.6 years. Two groups showed no significant differences in terms of gender, age, BMI, or comorbidities (diabetes and hypertension) (*P* > 0.05).

**Table 1:** General characteristics of the patients

General data	Non-staghorn group	Staghorn group	$x^{2}$/t	*P*-value
N	71(35.3)	130(64.7)		
Gender			0.007	0.936
Male	22(10.9)	41(20.4)		
Female	49(24.4)	89(44.3)		
Age	49.11 ± 13.58	52.14 ± 11.38	-1.681	0.094
Hypertension	21(34.4)	40(65.6)	0.031	0.861
Diabetes	10(40.0)	15(60.0)	0.273	0.601
BMI	24.89 ± 3.49	25.44 ± 5.27	-0.797	0.427

### Comparison of metabolic characteristics

Regarding serum biomarkers, patients in the staghorn calculi group exhibited significantly lower blood calcium levels compared to the non-staghorn calculi group (2.27 ± 0.11 mmol/L vs. 2.31 ± 0.11 mmol/L, *P* = 0.013). Furthermore, the eGFR was significantly lower in the staghorn calculi group than in the non-staghorn calculi group (74.79 ± 30.50 vs. 87.19 ± 33.94 mL/min, *P* = 0.009). No significant differences were observed between the two groups in terms of the serum levels of magnesium, phosphorus, potassium, chloride, sodium, blood urea nitrogen (BUN), creatinine, uric acid, or PTH (*P* > 0.05). Analysis of the metabolic parameters of 24-hour urine sample indicated that urinary calcium excretion was significantly lower in patients with staghorn calculi than in those with non-staghorn calculi (3.20 ± 2.19 vs. 4.26 ± 2.70 mmol/d, *P* = 0.003). Conversely, no significant differences were detected between the two groups regarding urinary pH, sodium, potassium, phosphorus, chloride, uric acid excretion, or total urine volume (*P* > 0.05) (Table 2).

**Table 2:** Metabolic profiles of the patients

Metabolic indicators	Group		*P*-value
	Non-staghorn Group(*n* = 71)	Staghorn Group (*n* = 130)	
**Serum Biochemical Factors**			
Calcium (mmol/L)	2.31 ± 0.11	2.27 ± 0.11	0.017
Magnesium (mmol/L)	0.89 ± 0.08	0.88 ± 0.08	0.349
Phosphorus (mmol/L)	1.18 ± 0.21	1.19 ± 0.16	0.748
Potassium (mmol/L)	4.00 ± 0.42	3.94 ± 0.37	0.270
Chloride (mmol/L)	106.34 ± 2.79	106.97 ± 3.40	0.186
Sodium (mmol/L)	141.49 ± 2.08	141.51 ± 2.62	0.950
BUN (mmol/L)	5.78 ± 3.08	6.79 ± 4.33	0.083
Creatinine (umol/L)	89.03 ± 58.74	103.78 ± 66.50	0.119
eGFR (mL/min)	87.19 ± 33.94	74.79 ± 30.50	0.009
Uric acid (µmol/L)	328.56 ± 102.31	358.39 ± 102.80	0.050
PTH (ng/mL)	52.44 ± 27.52	56.67 ± 47.80	0.494
**24-Hour Urine**			
pH (urine)	6.51 ± 0.63	6.54 ± 0.63	0.736
Calcium (mmol/d)	4.26 ± 2.70	3.20 ± 2.19	0.003
Sodium (mmol/d)	148.60 ± 66.85	154.02 ± 68.84	0.591
Potassium (mmol/d)	30.95 ± 12.65	29.17 ± 11.21	0.306
Phosphorus (mmol/d)	16.28 ± 10.05	15.53 ± 6.82	0.530
Chloride (mmol/d)	126.42 ± 56.19	127.43 ± 56.61	0.903
Uric acid (µmol/d)	2523.39 ± 1056.46	2630.82 ± 1119.62	0.508
Volume (L/d)	2.10 ± 0.78	2.17 ± 0.84	0.532

### Univariate and multivariate logistic regression analyses

Before multivariable logistic regression analysis, multicollinearity diagnostics were performed for all candidate variables. No evidence of severe multicollinearity was identified (all VIFs < 5 and tolerance values > 0.2) (Table 3). Therefore, all candidate variables were retained for multivariable analysis. Univariate logistic regression analysis indicated that serum calcium, eGFR, and urinary calcium were associated with staghorn calculus formation (*P* < 0.05), whereas age, BUN, and serum uric acid showed no significant association (*P* > 0.05) (Table 4).

**Table 3:** Collinearity diagnostic analysis

Parameter	VIF	Tolerance	
Age	1.663		0.601
Serum calcium (mmol/L)	1.088		0.919
Serum BUN (mmol/L)	1.898		0.527
eGFR (mL/min)	2.894		0.346
Serum uric acid (µmol/L)	1.336		0.749
Urinary calcium (mmol/d)	1.300		0.769

**Table 4:** Univariate logistic regression analysis

Metabolic Indicator	Group		*P*-value
	Non-staghorn Group (*n* = 71)	Staghorn Group (*n* = 130)	
Age	49.1 ± 13.6	52.1 ± 11.4	0.093
Serum calcium (mmol/L)	2.31 ± 0.11	2.27 ± 0.11	0.017
Serum BUN (mmol/L)	5.78 ± 3.08	6.79 ± 4.33	0.082
eGFR (mL/min)	87.19 ± 33.94	74.79 ± 30.50	0.009
Serum uric acid (µmol/L)	328.56 ± 102.31	358.39 ± 102.80	0.050
Urinary calcium (mmol/d)	4.26 ± 2.70	3.20 ± 2.19	0.003

To investigate the factors associated with the formation of staghorn calculi, a multivariable logistic regression analysis was performed, including variables such as age, serum calcium, serum blood urea nitrogen (BUN), eGFR, serum uric acid, and urinary calcium. The results showed that lower serum calcium level was significantly associated with the formation of staghorn calculi (*P* = 0.025, OR = 0.032, 95% CI [0.002, 0.649]) (Table 5). Serum calcium was rescaled by dividing by 0.1 mmol/L to improve clinical interpretability. The odds ratio was calculated per 0.1 mmol/L increase in serum calcium. The recalculated results were (*P* = 0.025, OR = 0.71, 95% CI [0.530, 0.960]).

**Table 5:** Multivariate logistic regression analysis of independent risk factors for infectious stone formation

Parameter	Regression Coefficient	Standard Error	Wald	*P*-Value	OR (95%CI)
Age	0.014	0.017	0.764	0.382	1.015 (0.982,1.048)
Serum calcium	-3.434	1.532	5.027	0.025	0.032 (0.002,0.649)
Serum BUN	-0.025	0.064	0.153	0.696	0.975 (0.861,1.105)
eGFR	-0.004	0.009	0.194	0.659	0.996 (0.980,1.013)
Serum uric acid	0.002	0.002	1.605	0.205	1.002 (0.999,1.006)
Urinary calcium	-0.126	0.071	3.182	0.074	0.882 (0.768,1.013)

### Distribution of culturable bacteria from urine culture

Of 201 patients with infectious stones, 124 cases showed positive midstream urine culture results. *Proteus mirabilis* (42 cases, 33.9%) and *Klebsiella pneumoniae* (10 cases, 8.1%) were the two most frequently detected urease-producing bacteria, and *Escherichia coli* (41 cases, 33.1%) and *Enterococcus faecalis* (8 cases, 6.5%) were the two most prevalent non-urease-producing bacteria. Comparative analysis of the microbial distribution between the staghorn calculi group and the non-staghorn calculi group revealed a significantly higher detection rate of *Proteus mirabilis* among patients in the staghorn calculi group (31.5% vs. 2.4%, *P* = 0.001) (Table 6).

**Table 6:** Distribution of positive urine culture isolates

Bacteria	Group (%)			$x^{2}$\Fisher	*P*-value
	Non-staghorn Group	Staghorn Group	Total		
Proteus mirabilis	3 (2.4)	39 (31.5)	42 (33.9)	10.801	0.001
Escherichia coli	14 (11.3)	27 (21.8)	41 (33.1)	2.733	0.098
Klebsiella pneumoniae	5 (4.0)	5 (4.0)	10 (8.1)	3.626	0.057
Enterococcus faecalis	4 (3.2)	4 (3.2)	8 (6.5)	2.851	0.091
Pseudomonas aeruginosa	2 (1.6)	3 (2.4)	5 (4.0)	0.625	0.429
Ureaplasma	0 (0.0)	6 (4.8)	6 (4.8)	2.102	0.147
Morganella morganii	0 (0.0)	2 (1.6)	2 (1.6)	0.678	0.410
Staphylococcus saprophyticus	0 (0.0)	2 (1.6)	2 (1.6)	0.678	0.410
Candida albicans	1 (0.8)	1 (0.8)	2 (1.6)	0.678	0.410
Mycoplasma hominis	1 (0.8)	1 (0.8)	2 (1.6)	0.678	0.410
Providencia rettger Proteus vulgaris	0 (0.0) 0 (0.0)	1 (0.8) 1 (0.8)	1 (0.8) 1 (0.8)	0.336 0.336	0.562 0.562
Streptococcus agalactiae	0 (0.0)	1 (0.8)	1 (0.8)	0.336	0.562
Haemophilus parainfluenzae	1 (0.8)	0 (0.0)	1 (0.8)	3.024	0.082
Total	31 (25)	93 (75)	124 (100)	-	-

## Discussion

Urinary tract stones represent a global health issue, affecting 5%-10% of the world’s population. The incidence of urinary tract stones continues to rise due to changes in diet, lifestyle, and metabolic factors [14]. Infectious stones, a specific type of urinary tract stone, are the primary component of staghorn calculi [15]. Staghorn calculi, characterized by their complex structure and large stone burden, can lead to severe complications, such as pyonephrosis, perinephric abscess, and pyelonephritis, if not treated in a timely manner [16]. Current research on staghorn calculi predominantly focuses on treatment strategies, while studies investigating their serum and urinary metabolic characteristics, particularly in the context of infectious stones, remain relatively scarce [17]. Therefore, this study strictly adhered to the predefined inclusion and exclusion criteria and enrolled 201 patients with infectious stones to compare differences in metabolic parameters and distribution of urinary bacteria detected by culture between those with staghorn and non-staghorn calculi. Patients in the staghorn calculi group exhibited significantly lower serum and urinary calcium levels and lower eGFR values, indicating that staghorn calculi were associated with poorer renal function in this cohort. Urine culture analysis also revealed a significantly higher prevalence of *Proteus mirabilis* in the staghorn calculi group. Besides, multivariate logistic regression analysis identified low serum calcium level as an independent factor associated with the formation of infectious staghorn calculi. These findings suggest that differences in metabolic profiles and the distribution of culturable urinary bacteria are associated with staghorn calculi.

Due to their large volume and frequent involvement of multiple calyces, staghorn calculi readily cause urinary tract obstruction and persistent infection. Over time, urinary tract obstruction and infection progressively deteriorate renal function, potentially advancing to end-stage renal disease or life-threatening uremia [18]. International studies have also confirmed that staghorn calculi are not only associated with declining renal function but may also induce systemic uremia [19]. An analysis of data from more than 1,000 patients with xanthogranulomatous pyelonephritis revealed that approximately 69% suffered from concurrent renal or ureteral stones, among whom 48% presented with staghorn calculi [20]. The conventional view suggests that struvite stones themselves can reduce eGFR [21]. Our findings are consistent with those of previous reports, emphasizing the importance of preventing staghorn calculi formation in the maintenance of renal function.

Numerous previous studies have reported a close relationship between nephrolithiasis and metabolic abnormalities. For instance, calcium oxalate stones, the most common types of kidney stones, were shown to be associated with hypercalciuria and hyperoxaluria, while hyperuricemia was found to be a significant risk factor for the formation of uric acid stones [22]. However, metabolic studies focusing specifically on patients with infectious staghorn calculi remain relatively scarce. This study found that among patients with infectious stones, those with staghorn calculi exhibit lower serum and urinary calcium levels. The kidneys play a central role in calcium homeostasis, with approximately 98% of calcium ions being reabsorbed in the renal glomeruli [23]. Renal tubular reabsorption of calcium is enhanced to maintain systemic calcium balance in case of hypocalcemia, leading to a corresponding reduction in urinary calcium excretion and lower urinary calcium levels. From the perspective of calcium-phosphorus metabolism, a decrease in the serum levels of ionized calcium can stimulate parathyroid hormone secretion, thereby promoting urinary phosphate excretion. Phosphate reabsorption further diminishes in renal insufficiency, thereby increasing the risk of phosphate crystal formation [24].

Previous studies have shown that the formation of infectious stones is closely associated with recurrent infection with urease-producing bacteria [25]. *Proteus* species, *Klebsiella* species, and *Pseudomonas* species represent common urease-producing pathogens [11]. Although these pathogens are all urease producers, their urease activity levels vary significantly. Previous studies have suggested that *Proteus mirabilis* possesses particularly high urease activity [26], a view supported indirectly by this study. Our microbial distribution results revealed a significant enrichment of *Proteus mirabilis* in the staghorn calculi group, indicating that this bacterium may promote the formation of staghorn calculi.

The mechanism behind infectious stone formation is well-established: firstly, bacterial urease enzymatically hydrolyzes urea into ammonia and carbon dioxide, which subsequently generate ammonium ions and bicarbonate. Subsequently, these ions bind to cations in the urine to form carbonate apatite and magnesium ammonium phosphate (struvite). Carbonate apatite begins to crystallize at a urine pH value of ≥ 6.8, while struvite only precipitates at a pH value of > 7.2 [11]. The alkaline environment formed by urease-mediated urea hydrolysis accelerates the precipitation of calcium and magnesium ions, thereby reducing urinary calcium levels. Concurrently, renal impairment caused by bacterial infection disrupts calcium and phosphorus metabolism and reduces urinary phosphate reabsorption. These processes accelerate the growth of infectious stones and staghorn calculi, establishing a vicious cycle of “infection-nephrolithiasis.”

Notably, although the detection rate of *Proteus mirabilis* was higher in the staghorn calculi group, no significant difference was observed between the two groups in terms of urine pH. Several factors may explain this finding. Urine pH was assessed using a single measurement, which could be affected by diet, fluid intake, and sampling time and might not reflect the status of urinary pH in the long term. The buffering capacity of urine itself may also mask intergroup differences. Furthermore, previous studies have shown that biofilms produced by bacteria can establish an alkaline microenvironment [27], which may not be captured by routine urine sampling. These results do not rule out the role of urine pH in the formation of staghorn calculi. Prospective cohort studies are needed to collect long-term urinary pH data from patients for further validation.

The findings of this study suggest that hypocalcemia may be associated with the formation of infectious staghorn calculi. Hypocalcemia may lead to elevated urinary phosphate levels through the calcium phosphorus metabolism pathway; at the same time, hypocalcemia may also result in hypocalciuria by enhancing renal tubular reabsorption of calcium, thereby reducing the physicochemical stability of urine (e.g., diminishing the inhibitory effect of citrate on nephrolithiasis). Although alterations in calcium phosphorus metabolism could potentially influence the urinary environment, the present retrospective observational study was not designed to clarify the underlying mechanisms, and it did not assess urinary supersaturation indices (e.g., calcium oxalate or calcium phosphate supersaturation), which also limits the interpretation regarding stone crystallization mechanisms. Therefore, it remains unclear whether lower serum calcium directly contributes to staghorn calculus formation or merely reflects underlying metabolic or renal functional changes associated with more advanced disease. The observed difference in serum calcium levels between groups in this study was statistically significant; however, the absolute difference was small and approached the margin of error of routine biochemical assays. Therefore, the clinical significance of this finding should be interpreted with caution, and additional studies with larger sample sizes and external validation cohorts are needed to confirm the robustness of this association.

This study also had several limitations. First, due to the inherent constraints of its retrospective design, we could not fully account for the potential effects of factors such as dietary habits, vitamin D levels, and the timing of infection. Second, our microbial analysis was based solely on urine culture, which may fail to detect bacteria when the urinary bacterial load is low. In addition, urine culture cannot identify low-abundance species in cases of polymicrobial infection. Moreover, we did not conduct microbiological culture or sequencing of the stones themselves, limiting a comprehensive understanding of the internal microbial composition of the calculi and the role of polymicrobial infections. Therefore, prospective cohort studies with more comprehensive data are needed to address these limitations and validate our findings.

Another limitation pertains to the multivariable regression model. Although multicollinearity diagnostics did not indicate severe collinearity among the included variables, the sample size was relatively limited, and thus some degree of model instability cannot be completely ruled out. Furthermore, because serum calcium was analyzed as a continuous variable using its original measurement unit and varied within a relatively narrow physiological range, the resulting odds ratio should be interpreted with caution. Future studies with larger sample sizes and external validation are needed to confirm the reproducibility and clinical significance of this finding. In addition, all laboratory data were collected at a single time point, which cannot represent the patients’ long-term metabolic status, and this constitutes another limitation of our study.

In summary, this study suggests that decreased serum calcium levels and Proteus mirabilis infection may be associated with the formation of infectious staghorn calculi. However, due to the retrospective design of this study, causality cannot be established, and further prospective studies are needed to determine whether serum calcium plays a role in this process.

## Data Availability

The datasets used and analyzed during the current study are available from the corresponding author on reasonable request.

## Abbreviations

eGFR: estimated glomerular filtration rate
BUN: Blood urea nitrogen
PTH: Parathyroid hormone
BMI: Body mass index
